# Dynamic Responses and Vibration Control of the Transmission Tower-Line System: A State-of-the-Art Review

**DOI:** 10.1155/2014/538457

**Published:** 2014-07-03

**Authors:** Bo Chen, Wei-hua Guo, Peng-yun Li, Wen-ping Xie

**Affiliations:** ^1^Key Laboratory of Roadway Bridge and Structural Engineering, Wuhan University of Technology, P.O. Box 219, No. 122 Luoshi Road, Wuhan 430070, China; ^2^Guangdong Power Grid Corporation Co. Ltd., Guangzhou 510080, China

## Abstract

This paper presented an overview on the dynamic analysis and control of the transmission tower-line system in the past forty years. The challenges and future developing trends in the dynamic analysis and mitigation of the transmission tower-line system under dynamic excitations are also put forward. It also reviews the analytical models and approaches of the transmission tower, transmission lines, and transmission tower-line systems, respectively, which contain the theoretical model, finite element (FE) model and the equivalent model; shows the advances in wind responses of the transmission tower-line system, which contains the dynamic effects under common wind loading, tornado, downburst, and typhoon; and discusses the dynamic responses under earthquake and ice loads, respectively. The vibration control of the transmission tower-line system is also reviewed, which includes the magnetorheological dampers, friction dampers, tuned mass dampers, and pounding tuned mass dampers.

## 1. Introduction

The degradation of civil engineering structures due to harsh environment may lead to structural damage and failure, associated with the events such as member fracture, column buckling, and brace breakage [1, 2]. To be a kind of high-rise structure with small damping, overhead transmission tower-line systems are critical infrastructure for electrical power transmission and are used throughout the world [3]. Transmission tower-line systems are prone to the dynamic excitation, such as wind, earthquake, and iced shedding. As supporting structures of coupled tower-line systems, transmission towers have relatively complex structural geometries and present obvious nonlinear vibration associated with flexibility of transmission lines. In reality, there exists a strong interaction between the motion of the truss tower and that of the transmission lines subjected to dynamic loading, each of which has frequency-dependent stiffness properties, leading to rather complex dynamic behaviour [4–6]. The failure of the towers under dynamic loading has been documented in many literatures [7, 8]. Therefore, it is relevant to assess the dynamic performance of transmission tower-line systems considering both elastic and inelastic responses.

The interest in the ability to monitor and mitigate the dynamic responses of the transmission tower-line system is pervasive throughout the civil and electrical engineering communities. To examine the properties of the coupled transmission tower-line system, many theoretical and experimental investigations have been carried out during the past two decades. With regard to the approaches and techniques used for performance evaluation and disaster mitigation, they can be classified into two major categories: one is the conventional approach without considering nonlinear tower-line interaction and the other is the approach based on coupled tower-line system. Conventionally, transmission tower-line systems can be designed and constructed using appropriate design standards [9–11]. The suggested design loads are commonly calibrated based on the assumption that the tower behaves elastically during dynamic excitation. In addition, the dynamic interaction between the tower and transmission lines cannot be taken into consideration during the common design process. Therefore, this design approach does not provide deep insights into inelastic and nonlinear tower behaviour under strong dynamic excitations, even though the consideration of inelastic responses can be important [12]. Furthermore, the primary environmental load considered in the design of transmission structures is the wind load, although the ice load may govern the design of transmission tower-line systems in some cold regions. Therefore, the damage and failure of transmission tower-line systems have been frequently reported across the world, even though the towers are designed and constructed strictly based on the specifications and codes.

After that, the development and application of structural assessment and mitigation approaches for transmission tower-line systems in the fields of civil and electrical engineering have attracted more and more attention. To overcome the shortcomings of conventional approaches, many analytical models and approaches have been proposed and developed for transmission tower-line systems in recent years with the aid of various techniques such as wind engineering, earthquake engineering, structural health monitoring, and vibration control. However, there are still many challenges and difficulties in the performance evaluation and vibration control techniques for the practical application of transmission tower-line system in various service conditions. Therefore, it is still essential to investigate the feasibility, validity, and applicability of the performance assessment and control approaches of the transmission tower-line systems.

This paper reviews the dynamic responses and control of the transmission tower-line system in the last two decades. The challenges and future trends in the disaster monitoring and mitigation of the transmission tower-line system subjected to dynamic excitations are also put forward. The structure of the rest of the paper is as follows.  Section 2 reviews the analytical models of transmission lines, truss towers, and the coupled tower-line system, which contains the theoretical model, finite element (FE) model, and the equivalent model; Section 3 reviews the wind responses of the transmission tower-line system, which contains the structural performance subjected to various wind loadings, such as common winds, tornado, downburst, and typhoon, respectively, and the experiment and field testing on wind effects; Sections 4 and 5 discuss the seismic responses and ice-induced responses of the transmission tower-line system, respectively. The vibration control of the transmission tower-line system is also reviewed. Finally, the challenges and future trends in the dynamic assessment and mitigation of transmission tower-line system are summarized in the conclusions.

## 2. Model of Transmission Tower-Line System

### 2.1. Model of Transmission Line

(*1) Theoretical Model.* To examine the properties of a coupled transmission tower-line system, many analytical models are developed and presented during the past two decades [13–16]. Irvine [13] systematically investigated the cable vibration through theoretical deduction and corresponding results are commonly taken as the benchmark to assess the effectiveness of various numerical simulating approaches. Based on the conclusions provided by Irvine [13], the natural frequencies of a transmission line for antisymmetric in-plane vibration *ω*_*s*_ can be expressed as

(1) $$\begin{array}{c} \omega_{s}=\frac{2n\pi }{l}\sqrt{\frac{H}{m}}\;\left(n=1,2,3,\ldots \right). \end{array}$$

The natural frequencies of a transmission line for the symmetric in-plane vibration can be determined by solving the following equations:

(2) $$\begin{array}{c} tg\frac{\eta }{2}=\frac{\eta }{2}-\frac{4}{\lambda^{2}}{\left(\frac{\eta }{2}\right)}^{2};\;\eta =\frac{\omega_{s}l}{\sqrt{H/m}};\;\;\lambda^{2}=\frac{{\left(mgl\right)}^{2}}{H^{3}}EA, \end{array}$$

where *H* is the tensile force of a transmission line; *m* is the mass of a transmission line per meter; *E* and *A* are the Young modulus and sectional area of a transmission line; *l* is the horizontal span of a transmission line. In addition, the natural frequencies of a cable for out-of-plane vibration *ω*_*v*_ are

(3) $$\begin{array}{c} \omega_{v}=\frac{n\pi }{l}\sqrt{\frac{H}{m}},\;\left(n=1,2,3,\ldots \right). \end{array}$$

(*2) FE Model.* A transmission line can be modelled by using cable elements in the FE method [17–19]. The equilibrium equation of the *i*th cable element can be established by using the virtual work principle based on the nonlinear FE method. The strain matrix of the *i*th cable element **B** ^(*i*)^ is the sum of the linear strain matrix **B**_*L*_^(*i*)^ and the nonlinear strain matrix **B**_NL_^(*i*)^:

(4) $$\begin{array}{c} B^{\left(i\right)}=B_{L}^{\left(i\right)}+B_{\text{NL}}^{\left(i\right)}. \end{array}$$

Both the linear strain matrix **B**_*L*_^(*i*)^ and the nonlinear strain matrix **B**_NL_^(*i*)^ relate to the shape function of a certain cable element. The stiffness matrix of the *i*th cable element **K** ^(*i*)^ in the global coordinate system (GCS) can be expressed as the sum of the elastic stiffness matrix **K**_*e*_^(*i*)^, displacement stiffness matrix **K**_*g*_^(*i*)^, and the stress stiffness matrix **K**_*σ*_^(*i*)^. Consider

(5) $$\begin{array}{c} K_{}^{\left(i\right)}=K_{e}^{\left(i\right)}+K_{g}^{\left(i\right)}+K_{\sigma }^{\left(i\right)}. \end{array}$$

**Figure 1:**
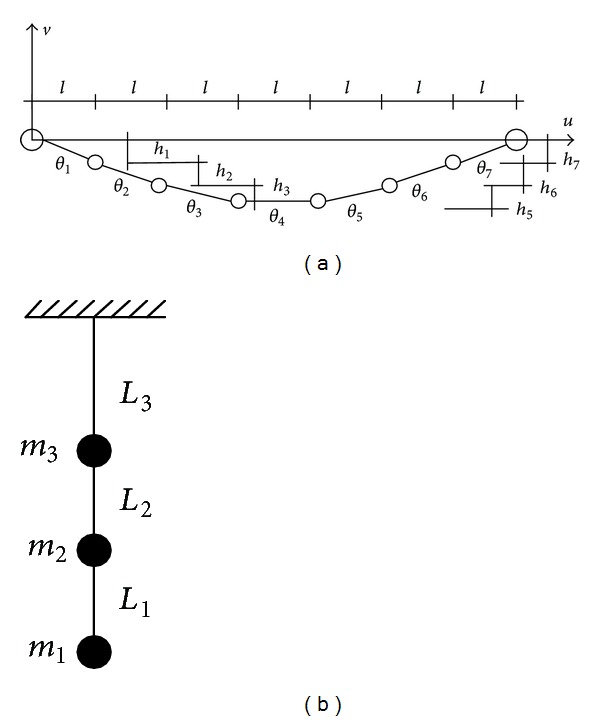
MDOF elastic model of a transmission line. (a) In-plane vibration. (b) Out-of-plane vibration.

The elastic stiffness matrix **K**_*e*_^(*i*)^ can be constructed only by the linear strain matrix **B**_*L*_^(*i*)^, while the displacement stiffness matrix **K**_*g*_^(*i*)^ can be constructed by both the linear strain matrix **B**_*L*_^(*i*)^ and nonlinear strain matrix **B**_NL_^(*i*)^. The stress stiffness matrix **K**_*σ*_^(*i*)^ is constructed by using the shape function of the cable element and the element stress *σ*. The global stiffness matrix of a transmission line can be determined by combining all the element stiffness matrices in the GCS:

(6) $$\begin{array}{c} K_{l}=\overset{nl}{\underset{i=1}{\sum }}K_{}^{\left(i\right)}, \end{array}$$

where *nl* denotes the number of all the cable elements in a transmission line. The mass matrix of the transmission line in the GCS can be expressed by using lumped mass matrix or consistent mass matrix based on the FE method. Consider

(7) $$\begin{array}{c} M_{l}=\overset{nl}{\underset{i=1}{\sum }}M_{}^{\left(i\right)}. \end{array}$$

(*3) MDOF Equivalent Model.* The transmission line can be simulated as several lumped masses connected with elastic elements as shown in Figure 1, which is the MDOF equivalent model. The Hamilton variational statement of dynamics indicates that the sum of the time variations of the difference in kinetic and potential energies and the work done by the nonconservative forces over any time interval *t*_1_ to *t*_2_ equals zero [19]. The application of this principle can lead directly to the equation of motion of a transmission line:

(8) $$\begin{array}{c} \overset{t_{2}}{\underset{t_{1}}{\int }}\delta \left[T_{\text{line}}\left(t\right)-U_{\text{line}}\left(t\right)\right]dt+\overset{t_{2}}{\underset{t_{1}}{\int }}\delta W_{\text{line}}\left(t\right)dt=0, \end{array}$$

in which *T*(*t*) and *U*(*t*) are the kinetic energy and potential energy of a transmission line. *W*_line_(*t*) equals the virtual work done by the nonconservative forces on a transmission line. It is clear that the transmission line may vibrate around its balanceable position when it is subjected to the external disturbance. The generalized coordinate *q*_*i*_ of a transmission line, namely, *ξ* and *δ*, can be defined as the difference of the angle *θ* and length *l*, respectively, as follows:

(9) $$\begin{array}{c} \xi_{i}=\delta \theta_{i}=\theta_{i}-\theta_{i0},\\ \delta_{i}=\delta l_{i}=l_{i}-l_{i0}-l_{is}, \end{array}$$

where *θ*_*i*0_ is the original value of *θ*_*i*_ for the *i*th element, *l*_*i*0_ and *l*_*i*_ are the original length and current length of the *i*th element, respectively, and *l*_*is*_ is the static deformation due to the gravity of the *i*th element.

The equation of motion of an N-DOF transmission line can be derived directly from the Hamilton equation by simply expressing the total kinetic energy *T*_line_, the total potential energy  *U*_line_, and the total virtual work *W*_line_ in terms of a set of generalized coordinates *q*_*i*_, namely, *ξ* and *δ*. Then, introducing the expression into the Hamilton equation and completing the variation of the first term yield the Lagrange equations of a transmission line as follows:

(10) $$\begin{array}{c} \frac{d}{dt}\left(\frac{\partial T_{\text{line}}}{\partial {\dot{q}}_{i}}\right)-\frac{\partial T_{\text{line}}}{\partial q_{i}}+\frac{\partial U_{\text{line}}}{\partial q_{i}}=Q_{i}, \end{array}$$

where *Q*_*i*_ is the generalized forcing function of the transmission line corresponding to the generalized coordinates *q*_*i*_.

After establishing the kinetic energy and potential energy of transmission line, the mass and stiffness matrices can be determined through partial differential calculation of the generalized velocity and generalized displacement, respectively. The mass matrix of a transmission line for the in-plane vibration **M**_*l*_^in^ can be deduced by computing partial differential of the derivative of generalized coordinates $\partial T/\partial {\dot{\xi }}_{i}$ and $\partial T/\partial {\dot{\delta }}_{i}$, respectively. The stiffness matrix of a transmission line for the in-plane vibration **K**_*l*_^in^ can be determined by computing partial differential of the generalized coordinates ∂*U*/∂*ξ*_*i*_ and ∂*U*/∂*δ*_*i*_, respectively. In addition, the transmission line can be simplified as a hanging line with a few lumped masses when considering the out-of-plane vibration. The mass matrix **M**_*l*_^out^ and stiffness matrix **K**_*l*_^out^ of transmission line can be deduced in the same way.

### 2.2. Model of Transmission Tower

(*1) FE Model.* The transmission tower is a typical spatial structure constructed by using steel members, which can be modelled by using beam and truss elements based on the FE method. The element stiffness matrix **K** ^(*m*)^ and mass matrix **M** ^(*m*)^ of the *m*th element in the GCS can be determined by transforming the element stiffness matrix **K**_*e*_^(*m*)^ and mass matrix **M**_*e*_^(*m*)^ in the local coordinate system (LCS) with the aid of coordinate transformation matrix **T**_*a*_^(*m*)^:

(11) $$\begin{array}{c} K_{}^{\left(m\right)}=T_{a}^{(m)T}K_{e}^{\left(m\right)}T_{a}^{\left(m\right)},\\ M_{}^{\left(m\right)}=T_{a}^{(m)T}M_{e}^{\left(m\right)}T_{a}^{\left(m\right)}. \end{array}$$

**Figure 2:**
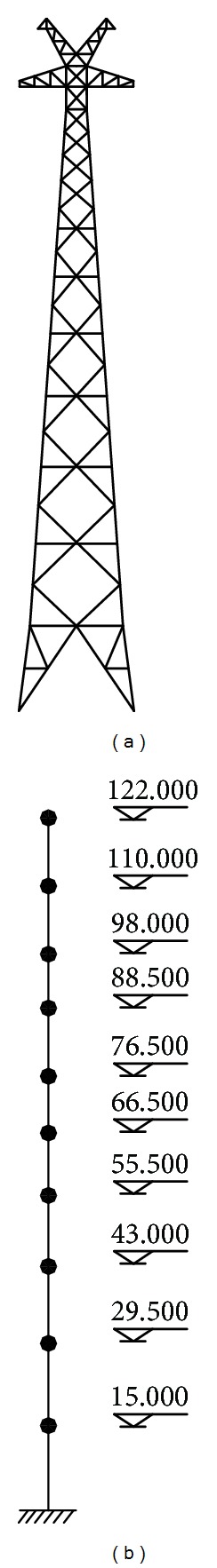
Analytical model of a transmission tower. (a) 3D FE mode. (b) 2D model.

After determining the element stiffness and mass matrices under the GCS, one can construct the position matrix of element freedom **T**_*c*_^(*m*)^ following the FEM connection information of each element under both local and global coordinate systems. Thus, the global stiffness matrix **K**_*t*_ and mass matrix **M**_*t*_ of a transmission tower in the GCS can be expressed as

(12) $$\begin{array}{c} K_{t}=\overset{ne}{\underset{m=1}{\sum }}T_{}^{(m)T}K_{}^{\left(m\right)}T_{}^{\left(m\right)},\\ M_{t}=\overset{ne}{\underset{m=1}{\sum }}T_{}^{(m)T}M_{}^{\left(m\right)}T_{}^{\left(m\right)}, \end{array}$$

where *ne* is the total element number of the finite element model of a transmission tower and **T** ^(*m*)^ is the freedom transform matrix from element coordinate system to the GCS, which is the product of coordinate transformation matrix **T**_*a*_^(*m*)^ and position matrix **T**_*c*_^(*m*)^ of the *m*th element.

(*2) 2D Lumped Mass Model.* If a 3D finite element dynamic model is used to model a tower with many transmission lines, the numerical step-by-step integration in the time domain to determine dynamic responses of the tower-line coupled system will be very time-consuming, which makes it impractical for parametric study and vibration control investigation. The dynamic excitation on the tower such as wind loads and earthquakes can usually be modeled as a stationary or nonstationary stochastic process in time and nonhomogeneous in space. The digital simulation of dynamic loading of a 3D finite element model of the transmission tower-line system with the aid of the spectral representation method [20, 21] may need enormous computation effort. To this end, a 2D lumped mass model is commonly used in practice to investigate the wind/earthquake-induced dynamic response of a complicated transmission tower-line system [22] (see Figure 2).

When a 3D FE dynamic model of a transmission tower is reduced to a 2D lumped mass model, some assumptions are commonly adopted. Firstly, the mass of the transmission tower, including the masses of all structural components and all nonstructural components and all equipment in the tower, is concentrated at several floors only. Then, the average of the displacements of all nodes at a given floor in one common direction is defined as the nominal displacement of that floor in that direction. Finally, only the horizontal dynamic loading and responses are considered.

With these assumptions, the number of dynamic degrees of freedom of a transmission tower in the lumped mass model is the number of floors selected. The mass matrix **M**_*t*_ of the lumped mass model is a diagonal matrix. The stiffness matrix **K**_*t*_ of the lumped mass model of *n* degrees of freedom can be obtained based on the 3D FE model of the transmission tower by taking the following steps: (1) apply the same horizontal force at each node at the *i*th floor such that the sum of all forces equals 1; (2) determine the horizontal displacement of each node at the *j*th floor and define the nominal displacement of the *j*th floor to have the flexibility coefficient *d*_*ji*_  (*i*, *j* = 1, 2,…, *n*); (3) form the flexibility matrix **F** of *n* × *n* dimension; (4) inverse the flexibility matrix to obtain the stiffness matrix **K**_*t*_.

### 2.3. Model of Transmission Tower-Line System

(*1) FE Model.* Similar to the construction process of a transmission tower, the global stiffness and mass matrices of a transmission tower-line system in the GCS can be established by combining the stiffness and mass matrices of towers and lines in the GCS by using the FE method:

(13) $$\begin{array}{c} K=\overset{n\text{tower}}{\underset{i=1}{\sum }}K_{t}^{\left(i\right)}+\overset{n\text{line}}{\underset{j=1}{\sum }}K_{l}^{\left(j\right)},\\ M=\overset{n\text{tower}}{\underset{i=1}{\sum }}M_{t}^{\left(i\right)}+\overset{n\text{line}}{\underset{j=1}{\sum }}M_{l}^{\left(j\right)}, \end{array}$$

where *n*tower and *n*line are the numbers of towers and transmission lines in a transmission tower-line system, respectively.

(*2) MDOF Equivalent Model.* As discussed above, the analytical model of a transmission tower-line system constructed by using the 3D tower model and the cable model may be very complicated and time-consuming in the numerical computation. Therefore, a MDOF equivalent model of the transmission tower-line system can be developed by combining the 2D tower model and the equivalent line model.

For the transmission tower-line system, the kinetic energy can be expressed in terms of the generalized coordinates and their first time derivatives, and the potential energy can be expressed in terms of the generalized coordinates alone. In addition, the virtual work which is performed by the nonconservative forces as they act through the virtual displacements caused by an arbitrary set of variations in the generalized coordinates can be expressed as a linear function of those variations. In mathematical terms the above three statements are expressed in the form

(14) $$\begin{array}{c} T=T\left(q_{1},q_{2},\ldots ,q_{N},{\dot{q}}_{1},{\dot{q}}_{2},\ldots ,{\dot{q}}_{N}\right),\\ V=V\left(q_{1},q_{2},\ldots ,q_{N}\right),\\ \delta W_{nc}=Q_{1}\delta q_{1}+Q_{2}\delta q_{2}+\cdots +Q_{N}\delta q_{N}, \end{array}$$

where the coefficients *Q*_1_, *Q*_2_,…, *Q*_*N*_, are the generalized forcing functions corresponding to the coordinates *q*_1_, *q*_2_,…, *q*_*N*_, respectively.

**Figure 3:**
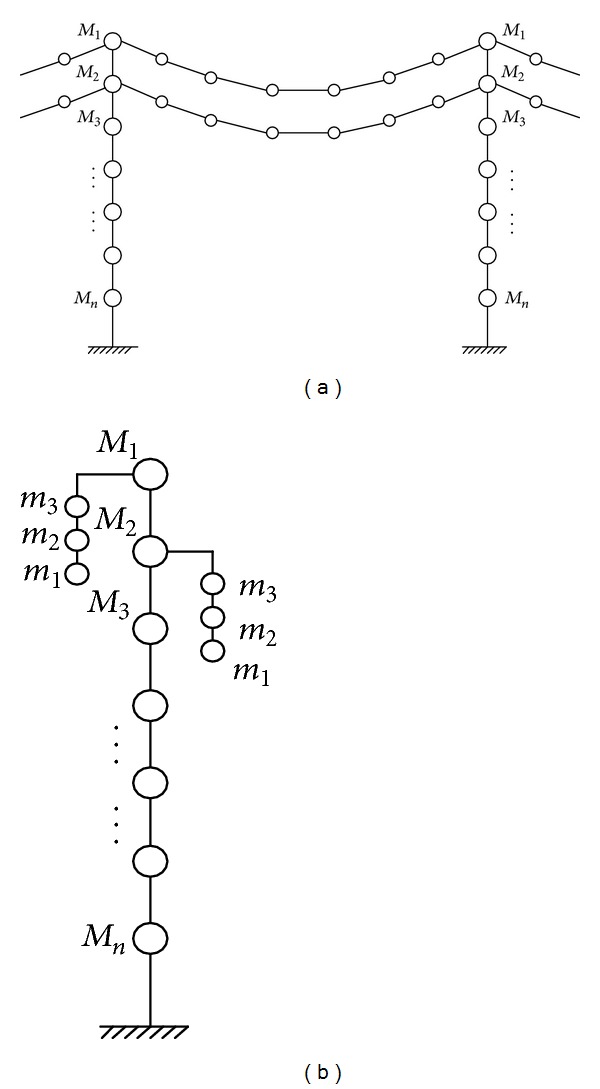
Analytical model of a transmission tower-line system. (a) In-plane vibration. (b) Out-of-plane vibration.

The analytical model of transmission tower-line system is displayed in Figure 3. The kinetic energy *T* and potential energy *U* of the coupled system are

(15) $$\begin{array}{c} T=\overset{n\text{tower}}{\underset{i=1}{\sum }}T_{t}^{\left(i\right)}+\overset{n\text{line}}{\underset{j=1}{\sum }}T_{l}^{\left(j\right)},\\ U=\overset{n\text{tower}}{\underset{i=1}{\sum }}U_{t}^{\left(i\right)}+\overset{n\text{line}}{\underset{j=1}{\sum }}U_{l}^{\left(j\right)}. \end{array}$$

By substituting (15) into the Lagrange equation, the motion of equation of a transmission tower-line system can be determined by computing the partial differential of the kinetic energy *T* and potential energy *U* to generalize coordinates and their first time derivatives.

## 3. Wind Responses of Transmission Tower-Line System

Transmission tower connected by many lines has more complex structural geometries and behaviour than common self-supported towers. Transmission tower-line system is a typical wind sensitive structure and wind loading often controls the structural design of transmission tower-line system [20, 21]. The response of structures to wind action may involve a wide range of structural actions, including resultant forces, bending moments, cable tensions, and deflections and acceleration. The transmission lines, being relatively slack under dead load, together with the behaviour of the tower and the conductors make the system very nonlinear. It was considered that since time history analysis takes into account nonlinearity this analysis is more accurate than the multimodal spectral analysis.

### 3.1. Performance Subjected to Common Wind Loading

Early studies on guyed towers for transmission lines were focused on the galloping phenomenon [23, 24]. Later works on the dynamic wind loading for transmission tower-line system, for example, the studies of Yasui et al. [25] and Battista et al. [26], did not involve flexible-type structures such as guyed towers. Liew and Norville [27] presented a method for studying the response of a transmission tower structural system subjected to wind loads. The wind speeds and the loads from the conductors were considered as the loadings on the transmission tower structural system. The data were used to determine the frequency response functions of the transmission tower structural system which provided a measure of response. Yasui et al. [25] described a method for analyzing wind-induced vibrations of power transmission towers coupled with power lines. They also discussed the influence on the response characteristics of differences in transmission support systems and the differences between peak factors, computed from a time series and from the power spectrum density. Battista et al. [26] proposed a new analytical-numerical modelling for the structural analysis of transmission line towers under wind action for stability assessment in a design stage. A simplified two-degree-of-freedom analytical model is also presented and shown to be a useful tool for evaluating the system fundamental frequency in early design stages. Loredo-Souza and Davenport [28] examined the influence of the design methodology in the response of transmission towers to wind loading. The Davenport gust response factor was compared with the statistical method using influence lines. From the results it can be concluded that the incorporation of the dynamic properties of transmission structures in the design methodologies is needed and that the statistical method using influence lines is a more correct approach since it allows for the inclusion of a larger number of factors in the design methodology.

The transmission tower-line systems become important infrastructures in modern societies and their wind-induced responses are an essential and practical task in the safety assessment. Okamura et al. [29] carried out the wind response analysis of a transmission tower in a mountainous area based on full-scale measurements. The wind response analysis results for the blowdown flow on the leeward slope of the mountain corresponded closely with the measurements. The analytical results demonstrate that the evaluation of the blowdown angle is also important in the wind response analysis of the transmission tower in the mountainous area. Liu and Li [30] presented an analytical framework to evaluate the along-wind-induced dynamic responses of a transmission tower. Two analytical models and a new method were developed. One was a higher mode generalized force spectrum model of the transmission tower and the other was an analytical model that includes the contributions of the higher modes derived as a rational algebraic formula to estimate the structural displacement response. A new approach was developed by applying load with displacement (ALD) instead of force to solve the internal force of transmission tower. It was found that the ALD method can avoid calculating equivalent static wind loads compared with conventional methods. The importance of the dynamic response of guyed towers for transmission lines under wind loading was evaluated by Gani and Légeron [31]. The research objective was to verify if the simplified static-equivalent approach provided in the current transmission line codes is sufficient for this type of flexible tower. It was found that the static-equivalent approach may underestimate the possible dynamic response. Similar investigations on wind-induced dynamic responses were carried out by Hou et al. [32] and Li et al. [33].

The numerical simulation of transmission tower-line systems' progressive collapse performance is considered as a major research hotspot and significant project, due to the increasing number of wind-induced collapse accidents recently. To assess the collapse risk of transmission line structures subject to natural hazards, it is important to identify what hazard may cause the structural collapse. Zhang and Li [34] introduced a new method termed as the probability density evolution method (PDEM) so as to accurately compute the dynamic response and reliability of a transmission tower. The random parameters of the wind stochastic field, such as the roughness length, the mean wind velocity, and the probability density functions, were investigated. It was found that not only the statistic quantities of the dynamic response, but also the instantaneous probability density function of the response and the time-varying reliability can be determined based on the proposed method. The results demonstrated that the PDEM is feasible and efficient in the dynamic response and reliability analysis of wind-excited transmission towers.

**Figure 4:**
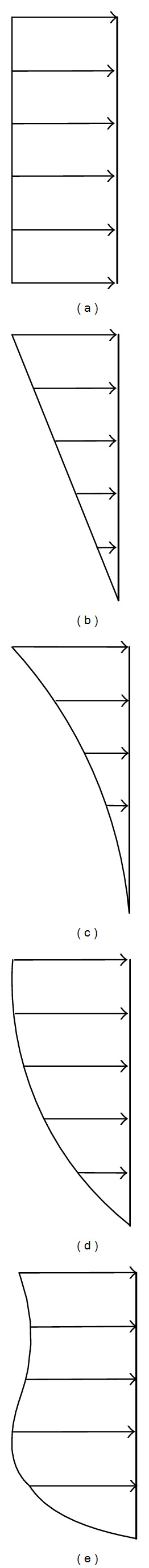
Load patterns for performance analysis of transmission tower: (a) rectangular, (b) inverted triangular, (c) first mode, (d) power law, and (e) tornado.

Banik et al. [35] assessed capacity curves for transmission line towers under wind loading. The assessment was performed by using a nonlinear static pushover (NSP) analysis and incremental dynamic analysis (IDA) using different load patterns as shown in Figure 4. For the IDA, temporally and spatially varying wind speeds were simulated based on power spectral density and coherence functions. Numerical results indicated that the structural capacity curves of the tower determined from the NSP analysis depend on the load pattern and that the curves determined from the nonlinear static pushover analysis were similar to those obtained from IDA. Furthermore, Mara and Hong [36] investigated the inelastic response of a self-supported transmission tower under different wind events, including traditional atmospheric boundary layer wind and downburst wind, and for wind loading at different directions relative to the tower. The NSP analysis was used to obtain the capacity curve of the tower, defined by the force-deformation relationship, at each considered wind direction. The results indicated that the yield and maximum capacities vary with wind direction.

Fei et al. [37] presented a method to evaluate the structural status of transmission lines based on dynamic and stability analysis. A long-span transmission tower-line system in China with a span of 1083 m was taken as the real example. Nonlinear buckling analysis for both the tower and tower-line systems was performed to determine the critical wind loads. Numerical results indicated that modal frequencies of low order modes decrease when the wind velocity increases before the structural instability happens in both cases. Therefore, for the structural health monitoring of transmission lines, frequency decrease of low order mode is a useful indicator to predict the happening of structural instability. Zhang et al. [38] examined wind-induced collapsed performance of a transmission tower-line system through numerical simulation. The finite element models for the single tower and transmission tower-line system were established to simulate wind-induced progressive collapse by using birth-to-death element technique with the aid of the commercial package ABAQUS. It is demonstrated that the collapse mechanism of the transmission tower-line system depended on the number, position, and last deformation of damage elements.

**Figure 5:**
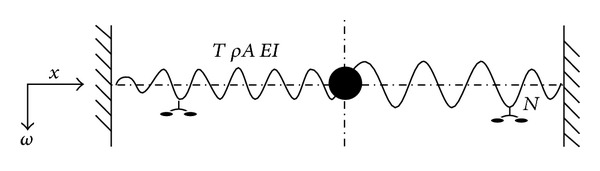
Schematic view of a typical long-span transmission line.

Galloping of overhead transmission lines has been under investigation for a long time in the industrial aerodynamics field and is still awaiting solution. It is important to understand the effects of wind turbulence on galloping and to establish an evaluation method for galloping of transmission line in gusty wind. Ohkuma and Marukawa [39] investigated the galloping of overhead transmission lines in gusty wind. They discussed the differences between galloping in smooth wind and galloping in gusty wind through a numerical simulation focusing on their behavior rather than their mechanisms. In addition, Verma and Hagedorn [40] developed a modified approach of the energy balance principle by taking into account in-span damping (Figure 5). The complex transcendental eigenvalue problem was solved for the conductor with in-span fittings. With the determined complex eigenvalues and eigenfunctions, a modified energy balance principle was then used for scaling the amplitudes of vibrations at each resonance frequency. Bending strains are then estimated at the critical points of the conductor.

### 3.2. Performance Subjected to Tornado

A thunderstorm, also known as an electrical storm, a lightning storm, thundershower, or simply a storm, is a form of turbulent weather characterized by the presence of lightning and its acoustic effect on the Earth's atmosphere known as thunder. Thunderstorms are usually accompanied by strong winds, heavy rain, and sometimes snow, sleet, hail, or no precipitation at all. There are several different types of thunderstorms, depending on the origin and the associated meteorological activities. All types of thunderstorms can occasionally become severe. The most severe thunderstorm is a tornado and another type of severe thunderstorm is the so-called downburst. In many countries, a large proportion of failures of transmission tower-line systems are caused by severe thunderstorms. Because the wind loads generated by thunderstorms are not only random but time-variant as well, a time-dependent structural reliability approach for the risk assessment of transmission tower-line system is essential. However, a lack of appropriate stochastic models for thunderstorm winds usually makes this kind of analysis impossible. To this end, Li [41] proposed a stochastic model to realistically and accurately simulate wind loading due to severe thunderstorms. With the proposed thunderstorm model, the collapse risk of transmission line structures under severe thunderstorms is assessed numerically based on the computed failure probability of the structure.

Tornadoes contain the most powerful effects of all winds [4]. A tornado consists of a vortex of air that develops within a severe thunderstorm and moves with respect to the ground with speeds of the order of 20–100 km/hr in a path. A tornado is a violently rotating column of air that is in contact with both the surface of the earth and the cumulonimbus cloud, which is often referred to as twister or cyclone. Tornadoes are observed as funnel-shaped clouds and the tangential speeds are probably highest at the funnel edge and drop-off toward the center and with increasing distance outside the funnel. Since the centrifugal forces in the tornado vertex far exceed the Coriolis forces, the latter may be neglected and the gradient wind equation can be expressed as

(16) $$\begin{array}{c} \frac{V^{2}}{r}=\frac{1}{\rho }\cdot \frac{dp}{dr}, \end{array}$$

where *V* is the cyclostrophic wind velocity, *r* is the radial distance from the center of the vortex, *ρ* is the air density, and the *dp*/*dr* is the pressure gradient along the radius. A tornado is different to downburst and microburst. In a tornado, high velocity winds circle a central point, moving inward and upward, whereas in a downburst the wind is directed downward and then outward from the surface landing point. Many transmission line and tower failures worldwide are attributed to high intensity winds associated with tornadoes.

Savory et al. [7] described models for the wind velocity time histories of transient tornado and microburst events and the resulting loads on a lattice transmission tower. A dynamic structural analysis was developed to predict a tornado-induced shear failure. The results from the predictions were encouraging in that the tornado failure appeared to concur well with evidence from the field, whilst the effect of the microburst was clearly less severe. Hamada et al. [42] developed a numerical scheme to assess the performance of transmission lines under tornado wind load events. The wind forces associated with these tornado fields were evaluated and later incorporated into a nonlinear finite element three-dimensional model for the transmission line system. A comparison was carried out between the forces in the members resulting from the tornadoes and those obtained using the conventional design wind loads. The study revealed the importance of considering tornadoes when designing transmission line structures.

Ahmed et al. [43] carried out the collapse and pull-down analysis of high voltage electricity transmission towers subjected to cyclonic wind. They presented a novel methodology developed for the critical infrastructure protection modelling and analysis (CIPMA) capability for assessing local wind speeds and the likelihood of tower failure for a range of transmission tower and conductor types. Similar work was conducted by Pecin et al. [44] to evaluate the mechanical global actions due to an approximate mathematical model of a tornado. Usage of tornadic response spectrum practices was proposed and particular aspects of tornadic loads on tower structures were analyzed.

### 3.3. Performance Subjected to Downburst

**Figure 6:**
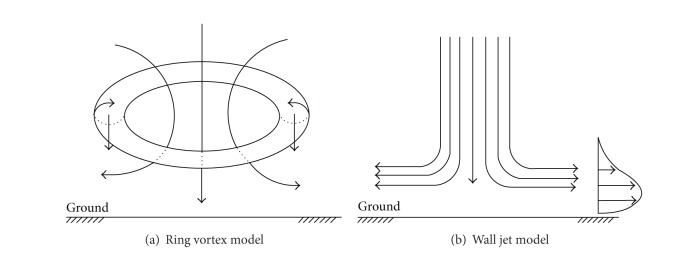
Typical models of downburst.

A downburst is a strong ground-level wind system that emanates from a single source, blowing in a straight line in all directions from that source. Downbursts are created by an area of significant rain-cooled air that after reaching ground level spreads out in all directions producing strong winds. Downbursts include microbursts and macrobursts [45]. Microbursts are smaller and more concentrated than downbursts, the physical size of which is about 4 km or less in horizontal extent. A macroburst is a large downburst. The physical size of thunderstorm activities in Australia is shown in Table 1 [46]. Downbursts can induce an outburst of damaging winds near the ground, with near surface speeds in excess of 50 m/s. During the past decade, many electrical transmission tower structures have failed during downburst. The nature of the loading imposed on a transmission tower by a downburst will depend upon the stage of the development of the event when it interacts with the tower [7]. If the downburst is close to the ground and approaching touchdown, then there may well be a significant vertical loading component on the tower. However, if the microburst has already reached the ground and is spreading outward as it impinges upon the tower, then the main loading components will be in the horizontal plane. There are essentially two forms of simplified models for the wind field associated with a downburst [47, 48], namely, the ring vortex model and the impinging wall jet model, as illustrated schematically in Figure 6. Many studies have been performed to understand the behavior of transmission tower-line system under such localized wind events.

Shehata et al. [49] assessed the effects of varying the downburst parameters on the performance of a transmission line structure by taking several real towers as examples, which were failed in Manitoba, Canada, during a downburst event in 1996. The spatial and time variation of the downburst wind field was examined. Then, the variations of the tower members' internal forces with the downburst parameters were discussed. In addition, the structural behavior under critical downburst configurations was compared to that resulting from the boundary layer normal wind load conditions. Furthermore, they [50, 51] performed the failure analysis of a transmission tower that collapsed in Winnipeg, Canada, subjected to a microburst event. Their study was conducted using a fluid-structure numerical model that was developed in-house. The model was employed first to determine the microburst parameters that are likely to initiate failure of a number of critical members of the tower. Progressive failure analysis of the tower was then conducted by applying the loads associated with those critical configurations.

Darwish et al. [52] assessed the dynamic characteristics and behavior of transmission line conductors under the turbulent downburst loading. A nonlinear numerical model was developed and used to predict the natural frequencies and mode shapes of conductors at various loading stages. Dynamic analysis was carried out using various downburst configurations. The made observations indicated that the responses are affected by the background component, while their sonant component turns to be negligible due large aerodynamic damping of the conductors. Darwish and Damatty [53] also investigated the behavior of self-supported transmission line towers under downburst loading. A parametric study was performed to determine the critical downburst configurations causing maximum axial forces for various members of a tower. The sensitivity of the internal forces developing in the tower members to changes in the downburst size and location was studied. The structural behavior associated with the critical downburst configurations was described and compared to the behavior under “normal” wind loads.

### 3.4. Performance Subjected to Typhoon

The winds produced by severe tropical cyclones also known as “hurricanes” and “typhoons” are the most severe wind loading on earth. However, their infrequent occurrence at particular locations often makes the historical record of recorded wind speeds an unreliable predictor for design wind speeds. Bulk transmission tower-line system is prone to strong typhoon loadings, particularly at the open coastal terrain in cyclonic regions. The investigation on the performance of the transmission tower-line system subjected to typhoon is limited due to the difficulties in collecting typhoon wind loading.

Tomokiyo et al. [54] reported the typhoon damage analysis of transmission towers in mountainous regions of Kyushu, Japan. They have operated a network for wind measurement, NeWMeK, which measures wind speed and direction, covering these mountainous areas, segmenting the Kyushu area into high density arrays since 1995. In particular, they discussed the wind characteristics of Typhoon Bart in 1999 and the damage to towers located in the mountainous regions along with the distribution and direction of fallen trees. It was observed that transmission towers were damaged by winds that became stronger due to the effect of the local terrain or by being involved in changes in tensile forces of the transmission lines of the towers that had already collapsed. These towers were collapsed due to a combination of the above factors. The world tallest transmission tower, the 370 m Zhoushan transmission towers over the typhoon-prone sea strait, was taken as an example by Huang et al. [55] to examine structural wind effects. Time domain computational simulation approach was also employed to predict dynamic responses of the transmission tower and the displacement based gust response factors (GRFs). The fair comparison of gust loading factors or GRFs was made between the results of the experimental approach and the computational simulation approach, which was an effective alternative way for quickly assessing dynamic wind load effects on high-rise and complex tower structures.

### 3.5. Experiment and Field Testing for Wind Effects

**Figure 7:**
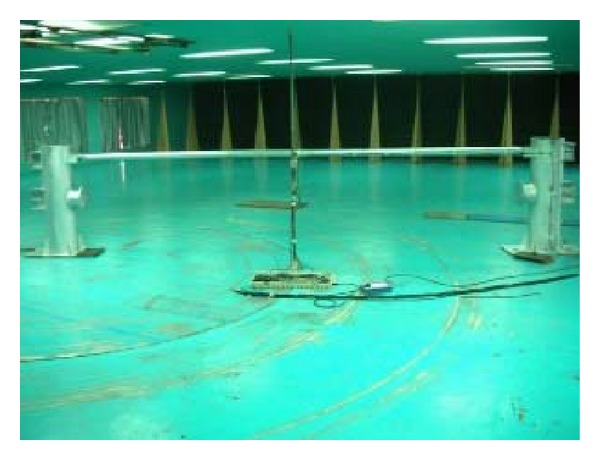
View of wind tunnel testing of the vortex-induced vibration.

**Figure 8:**
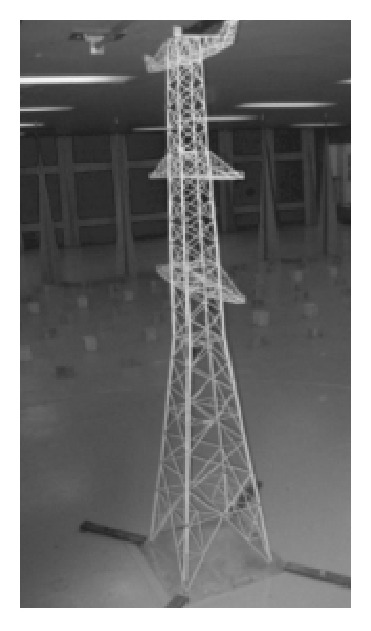
Scheme of the field testing.

(*1) Wind Tunnel Test.* Compared to the theoretical and numerical investigation, the studies on the performance of transmission tower-line system through experiments and field measurement are quite limited. Vortex-induced vibration is a critical problem for the steel cylinders used in tubular towers, such as transmission towers. Therefore, Deng et al. [56] performed vortex-induced vibration tests on lull-scale cylinders to study the vibration performance of steel tubes connected with typical joints in transmission towers, including [-shaped gusset plate connection, U-shaped gusset plate connection, cross-gusset connection, and the flange (see Figure 7). The testing observations indicated that vortex-induced vibration can occur not only in laminar flows, but also in turbulent flows, and the amplitude decreases as the turbulence intensity rises. In addition, Deng et al. [57] carried out the wind tunnel study on wind-induced vibration responses of an ultra-high-voltage (UHV) transmission tower-line system. A discrete stiffness method was applied to design the aeroelastic model on the basis of similarity theory as shown in Figure 8. The dynamic characteristics of the single tower and the tower-line system were identified and the displacement responses at different positions were obtained under a variety of wind speeds. It was found that the wind-induced vibration coefficient specified by the code is much smaller than that by testing. Thus, the code value seems to be unsafe for the UHV transmission tower.

**Figure 9:**
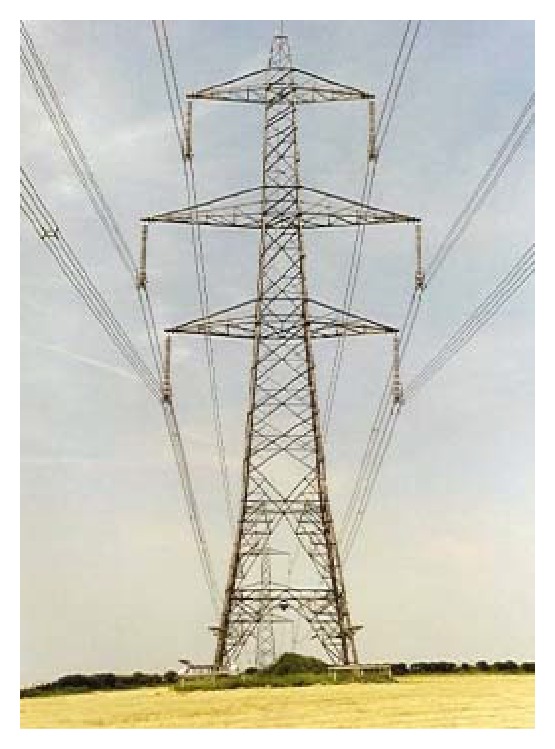
The monitored L6 transmission line tower.

Strong winds are observed commonly associated with heavy rains. The wind-rain-induced vibration and damage of civil engineering structures are frequently reported, in particular for cables and transmission lines. Li et al. [58] carried out the testing on wind-rain-induced vibration of transmission towers. The aeroelastic models of the antelope horn tower and pole tower were manufactured based on the similarity theory for the wind tunnel tests. The response analyses and experiments for the two kinds of models were conducted under the wind-induced and wind-rain-induced actions with the uniform and turbulent flow. It was shown that the results of wind-rain-induced responses were bigger than those of only wind-induced responses.

(*2) Field Testing.* Savory et al. [59] discussed some of the findings arising from long-term monitoring of the wind effects on a transmission tower located on an exposed site in South West England. Site wind speeds and foundation loads were measured. Comparisons between the measured strains and those determined based on UK code indicated that the code overestimates most of the measured foundation loads by a moderate amount of about 14% at higher wind speeds. This tends to confirm the validity of the code for assessing design foundation loads. Furthermore, Savory et al. [60] presented a comparison between the wind-induced foundation loads measured on a type L6 transmission line tower (see Figure 9) during a field study in the UK and those computed using the UK Code of Practice for lattice tower and transmission line design. The analysis demonstrated excellent agreement between the code calculations and the measured results.

**Figure 10:**
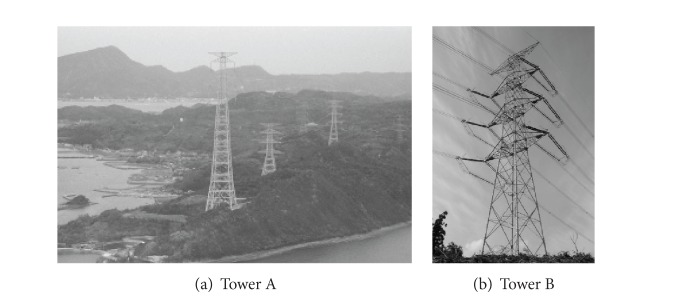
Elevation of the example towers.

The galloping is commonly observed in the overhead transmission line vibration during the ice storm. A method of single channel signal processing was implemented by Gurung et al. [61] to discuss galloping of transmission lines based on field data. Then, the same method was extended by them [62] to identify and characterize several numbers of vibrations observed in the Tsuruga Test Line of Kansai Electric Power Company during ice storms. The piecewise application of Prony's method was introduced to discuss time-dependent characteristics of harmonic components in the responses. The existence of motion-induced force was then confirmed for galloping events by introducing the usual buffeting theory. Based on full-scale measurement data, Takeuchi et al. [63] reported on several aerodynamic damping properties of two transmission towers under conditions of strong winds. They introduced a new method of estimating damping properties, which was applicable to the response record of a multidegree of freedom system such as the coupled structure of a transmission tower and conductors. The component of every vibration mode of the towers was extracted from a measured time history and the accurate damping ratios were estimated individually (see Figure 10).

## 4. Seismic Responses of Transmission Tower-Line System

**Figure 11:**
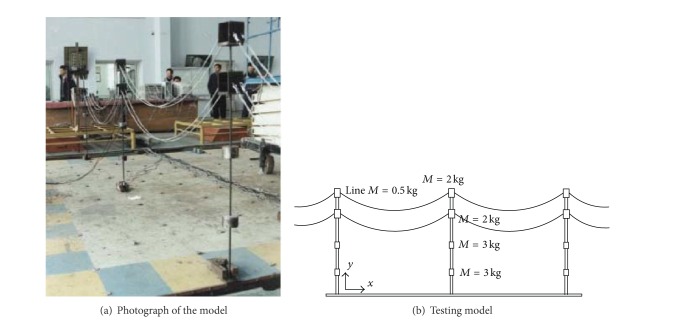
Elevation of testing model.

The conventional seismic assessment of transmission towers is usually carried out by considering each tower as an individual structure without taking the inertia coupling and the strong traction of transmission lines into consideration. In addition, many of structural engineers were used to simply ignore the wire mass or to simplify the transmission lines as a series of lumped masses affiliated to the tower in seismic computation. Up to now, the researches related to the seismic performance of transmission tower-line systems are limited. To this end, Li et al. [64] developed an analytical model for the seismic analysis of the transmission tower-line system by considering the tower-line interaction. To verify the validity of the proposed model, the shaking-table experiments of the coupled tower-line system were carried out as displayed in Figure 11. The results indicated that the errors of theoretical and testing results of systemic seismic responses are within the acceptable range. Based on the made observations, a simplified analysis method was proposed to make the seismic response calculation of coupled system faster and more effective.

Taniwaki and Ohkubo [65] developed an efficient optimal synthesis method to determine the optimum solutions for the structural shape, cross-sectional dimensions, and material type of all member elements of large-scale transmission towers subjected to static and seismic loads. The example of a cost-minimization problem for a real transmission tower that considers not only the material costs, but also the cost of land as objective functions was presented to demonstrate the rigorousness, efficiency, and reliability of the proposed method. Lei and Chien [66] investigated the dynamic behavior of transmission towers linked together through electrical lines when subjected to a strong ground motion. The transmission lines and the towers were modeled by using the cable elements and the 3D beam elements, respectively, both considering geometric nonlinearities. The strength capacities and the fracture occurrences for the main members of the tower were examined with the employment of the appropriate strength interaction equations. The made observation indicated that the ignorance of cable contribution to total seismic responses, especially the portion caused by the cable mass, would induce significant errors in predicting the ultimate strength of tower members. More recently, Wang et al. [67] carried out the progressive collapse analysis of the transmission tower-line system under earthquake with the aid of the commercial package ABAQUS. The collapse paths and failure positions of the power transmission tower were obtained under different seismic excitations.

Tian et al. [68] studied the seismic responses of the transmission tower-line system subjected to spatially varying ground motions. The towers were modeled by using beam elements and the transmission lines were modeled by using cable elements considering the nonlinear geometry. Both the incoherency of seismic waves and wave travel effects are taken into account. The effects of boundary conditions, ground motion spatial variations, incident angle of the seismic wave, coherency loss, and wave travel on the system were investigated in detail. The observations demonstrated that the uniform ground motion at all the support of the system cannot provide the most critical case for the response calculations of the transmission tower-line system. In addition, they [69] examined the dynamic responses of a transmission tower-line system at a canyon site under spatially varying ground motions. The spatially varying ground motions were simulated stochastically based on an empirical coherency loss function and a filtered Tajimi-Kanai power spectral density function. It was found that neglecting motion spatial variations may lead to a substantial underestimation of the responses of the transmission tower-line system during strong earthquakes. Furthermore, Li et al. [70] analyzed the effects of multicomponent multisupport excitations on the responses of a transmission tower-line system. Multicomponent and multisupport earthquake input waves were generated based on the code for the seismic design of electrical installations. An extensive parametric study was conducted to investigate the behavior of the transmission tower-line system. Similar investigations were conducted by Bai et al. [71] to study the nonlinear responses of a transmission tower-line system on a heterogeneous site subjected to multicomponent spatially varying ground motions. The made observations revealed that the multisupport and multicomponent earthquake excitations with consideration of the site effects should be considered in a reliable seismic response analysis of the transmission tower-line system.

## 5. Ice-Induced Response of Transmission Tower-Line System

**Figure 12:**
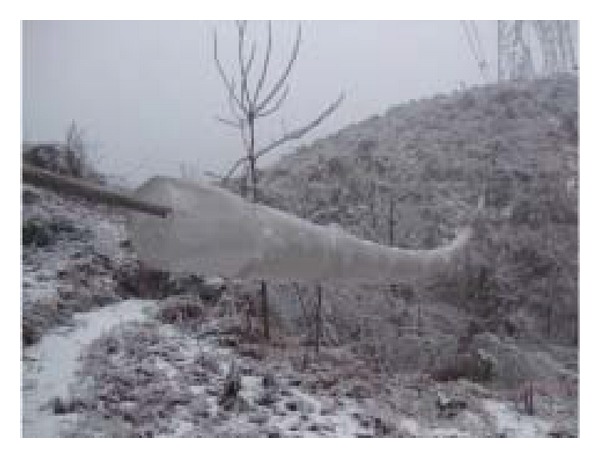
Accreted ice of the transmission line section.

Temperature load is a typical environmental loading acting on the civil engineering structures, in particular in some cold regions [72–74]. Ice load and its effects on transmission tower-line system have been substantially considered in the design, construction, and maintenance. Ice shedding can be observed when the transmission line and the conductor are subjected to the increasing environmental loading and dynamic excitations (see Figure 12). Shedding of the ice that accreted on transmission line cables is a common and practical issue in cold regions across the world. The falling of ice chunks may result in high-amplitude vibration of the deiced transmission lines and induce intensive dynamic forces [75]. Bundle collapse of a transmission line occurs when the bundle rotation exceeds a critical angle so that the bundle loses its stability [76, 77]. Ice shedding may easily induce electrical and mechanical accidents and thereby cause a serious damage to transmission tower-line system, which attracts more and more attention across the world. Havard and Dyke [78] reviewed ice-related dynamic problems on overhead lines, including ice shedding and bundle rolling.

Jamaleddine et al. [79] investigated the ice shedding from a two-span section using the commercial FE analysis software ADINA. They carried out a total of 44 tests on a reduced-scale two-span model to study the effects of ice shedding on overhead lines. Model predictions were validated on a small-scale laboratory model. McClure et al. [80, 81] studied the effects of ice thickness, partial shedding, and different line parameters on the dynamic response of ice shedding on transmission lines by a similar numerical approach. Jakše et al. [82] developed a numerical model to examine the ice-shedding effects of a 110 kV overhead power line in Slovenia. A single-span and three-span FE models of conductors were established in the computation. The made observations demonstrated that the deflected line configuration and large-amplitude oscillations resulting from load shedding were problematic. The situation was corrected by the utility on some line sections by installing interphase long insulating rod spacers. Kálmán et al. [83] established a nonlinear FE model for ground wires by ADINA, and several ice-shedding scenarios were studied with variables including span length and pulse-load characteristics. Kollár and Farzaneh [84] numerically examined the conductor vibration following ice shedding from one subconductor in a bundle. Furthermore, they [85] presented a different modeling approach to examine the dynamic behavior of a spacer damper located at midspan in twin, triple, and quad bundles after ice shedding.

**Figure 13:**
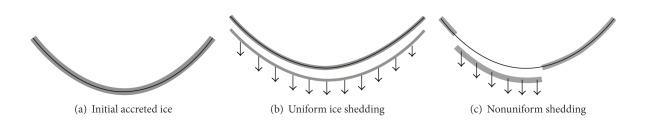
Ice-shedding scenarios.

Fengli et al. [86, 87] investigated dynamic responses of transmission tower-line system under ice shedding. The 3D FE model of a tower-conductor-wire-insulator system was established by using commercial package ANSYS, and the dynamic responses induced by the ice shedding were analyzed by considering different loading scenarios as shown in Figure 13. Many factors were considered in the ice-shedding simulations such as tower-line coupled effect, phase combination of the ice-shedding conductors, thickness of the accreted ice, length of the ice-shedding span, and elevation difference. Effects of different factors on the dynamic responses of jumping heights, loads at the end of insulators, and the forces of transmission tower were also studied. The made observation indicated that stress ratios of members at the tower head under design ice thickness exceed the permitted values under a large intensity of ice shedding. In addition, Yang et al. [88] also analyzed the unbalanced force of the transmission tower-line system in heavy icing areas. A seven-continuous-span conductor-string model of transmission lines was developed to examine the effects of design parameters, which included the loading mode of accreted ice, the eccentricity of accreted ice, the wind velocity, the ice thickness, the icing rate, the span length, the elevation difference, and the span difference.

**Figure 14:**
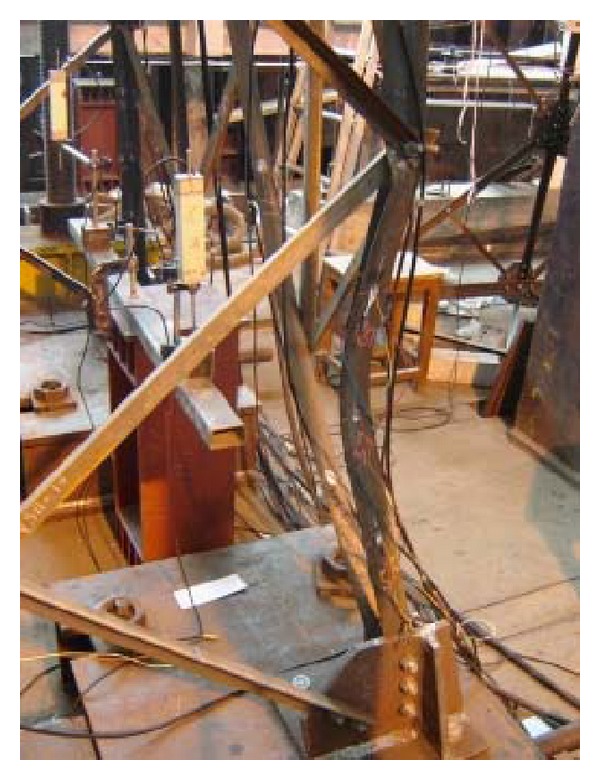
Failure phenomena of single-panel subassemblage without diaphragms.

Xie and Sun [89] studied the failure mechanism of transmission towers under ice loads and investigated the pertinent retrofitting strategy for increasing the load-carrying capacity of the tower. An experimental study was conducted on two pairs of subassemblages of a typical 500 kV transmission tower of the same type as those suffered the most severe damage during the ice disaster in South China in 2008 (see Figure 14). The mechanical behavior, failure mode, strain, and deformation at critical points of the specimens were studied. The made observations revealed that buckling of the main leg was the predominant failure mode of structures. It was found that the addition of the diaphragm significantly improved the mechanical performance of transmission towers by reducing the torsional effect on main members and inhibiting the out-of-plane deformation of diagonal braces.

Kollar and Farzaneh [90] investigated the ice shedding from conductor bundles through both numerical simulation and experiment. A FE model was developed to predict the transversal line motion as well as bundle rotation and to simulate shedding of concentrated loads. The experimental simulation was implemented by load shedding tests on a small-scale laboratory model. Numerical model predictions were validated by comparing them to observations obtained from experiments and full-scale tests. Yang et al. [91] carried out the analysis of the dynamic responses of a prototype line from iced broken conductors. A full-scale transmission line section of three continuous spans was established and steel cables were used to simulate the iced conductors by considering the equivalent mass of the accreted ice. Broken conductor experiments were carried out for different types of conductors and ice thickness. Time histories of the tensions and displacements at the middle of conductor spans were measured. The experimental results indicated that the impact effect is more significant for the location nearer to the break point. The dynamic impact factors decrease with the increase of the ice thickness, and the impact factors of conductors without accreted ice are much higher than those of conductors with accreted ice.

## 6. Vibration Control of Transmission Tower-Line System

**Figure 15:**
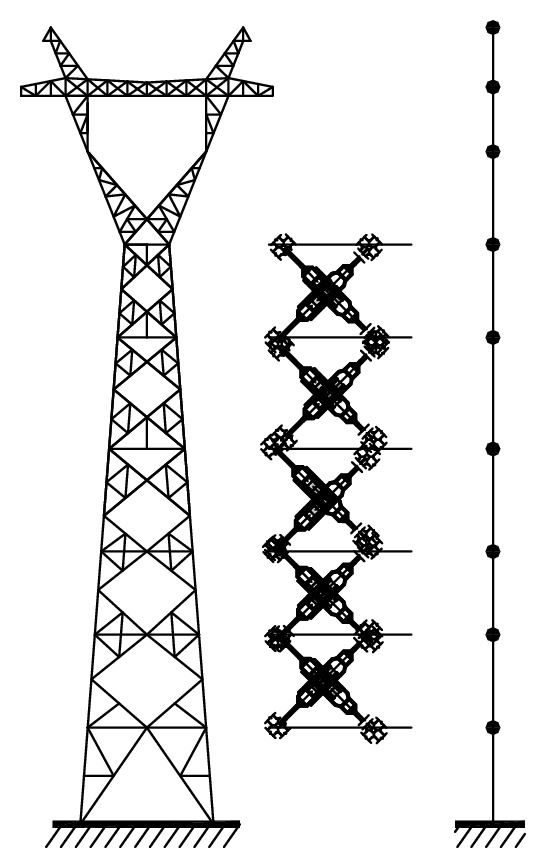
Installation scheme of energy-dissipating dampers on transmission tower.

Conventional disaster-resistant design of transmission tower-line system is based on the ductility of the structure that dissipates vibrating energy induced by dynamic excitations while accepting a certain level of structural damage. An alternative approach to prevent catastrophic damage of transmission tower-line system is to install control devices. Current studies on the vibration mitigation of transmission tower-line systems focus on the application of dynamic absorbers and energy-dissipating dampers. Different types of energy-dissipating dampers have been developed recently as an alternative approach for dynamic mitigation of transmission tower-line system. The dampers can be manufactured as an axial member to replace common structural members of a truss tower and, thus, it avoids the additional occupancy of structural space. Furthermore, passive and semiactive dampers can reduce dynamic responses of all mode shapes of the transmission tower-line system.  Figure 15 displays a typical installation scheme of energy-dissipating dampers in a transmission tower.

The equation of motion of the tower-line system with control devices subjected to dynamic excitations can be expressed as

(17) $$\begin{array}{c} M\ddot{x}\left(t\right)+C\dot{x}\left(t\right)+Kx\left(t\right)=P\left(t\right)+Hu\left(t\right), \end{array}$$

where **M**, **C**, and **K** are mass, damping, and stiffness matrices of the transmission tower-line system, respectively; **x**(*t*), $\dot{x}(t)$ and $\ddot{x}(t)$ are the displacement, velocity, and acceleration responses with respect to the ground, respectively; **P**(*t*) is the dynamic excitations; **u**(*t*) is the force provided by control devices for suppressing dynamic vibration; and **H** is the influence matrix for **u**(*t*).

**Figure 16:**
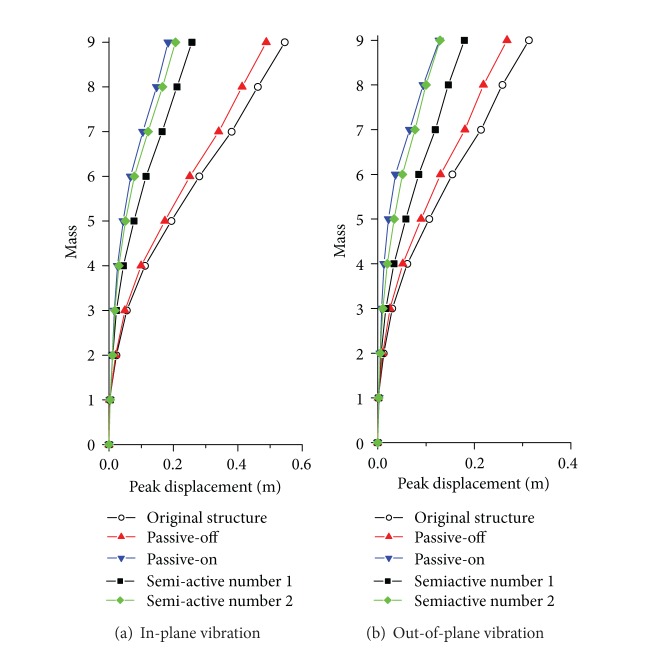
Comparison of control performance of peak displacement.

Different types of semiactive devices can be developed to equip control devices with actively controlled parameters forming a semiactive yet stable and low-power consuming damping system. Chen et al. [22, 92] firstly proposed a novel approach for the semiactive control of transmission tower-line system under dynamic excitations by using magnetorheological (MR) dampers. MR dampers are typical smart (semiactive) dampers and may overcome the shortcomings of dynamic absorbers because of their excellent control performance. A dynamic iteration process was developed for the numerical simulation of the dynamic responses of the transmission tower-line system. Two semiactive control strategies were proposed for the vibration mitigation of tower-line system. The first one was based on fixed increment of controllable damper force as expressed in

(18) $$\begin{array}{c} F_{d}\left(t+\Delta t\right)=F_{d}\left(t\right)+\alpha \cdot F_{d}\left(t\right),\;\left(\dot{d}\left(t\right)\neq 0\right),\\ F_{d}\left(t+\Delta t\right)=F_{d}\left(t\right)-\alpha \cdot F_{d}\left(t\right),\;\left(\dot{d}\left(t\right)=0\right), \end{array}$$

where *F*_*d*_(*t*) is the controllable Coulomb damping at time instant *t*, *α* is the increment coefficient of the damping force, and $\dot{d}(t)$ is the slipping velocity of MR damper at time instant *t*. The second one was a clipped-optimal strategy based on fuzzy control principle as expressed in

(19) $$\begin{array}{c} F_{d}\left(t\right)=\{\begin{array}{cc} \min\left[abs\left[K_{d}\left(x_{b}-e\right)\right]-F_{0},F_{\max}\right] & \\ \;\;\;\left(u\left(t\right)\cdot u^{f}\left(t\right)>0,\left|u^{f}\left(t\right)\right|>\left|u\left(t\right)\right|\right) & \\ F_{\min}\;\left(\text{other}\;\text{cases}\right), & \end{array} \end{array}$$

where *F*_0_ is a small adjustable quantity, *F*_max⁡_ and *F*_min⁡_ are the coulomb damper forces corresponding to the *τ*_*y*max⁡_ and *τ*_*y*min⁡_, respectively, and *u* ^*f*^(*t*) is the active control force determined based on fuzzy rules. A real transmission tower-line system constructed in Southern China was taken as an example to examine the feasibility and reliability of the proposed control approach. In addition, a parametric study was conducted in order to examine the effects of brace stiffness, wind loading intensity, and parameters of MR fluids on the control performance. The results as shown in Figure 16 demonstrate that the MR dampers can be utilized on the wind-induced vibration control of transmission tower-line system because of its simple configuration as well as its satisfactory energy-dissipating capacity if the damper parameters are optimally determined.

**Figure 17:**
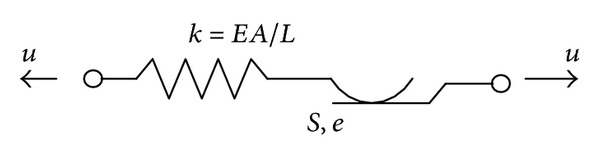
Mechanical model of a semiactive friction damper.

Chen et al. [93] proposed an integrated approach to realize both the vibration control and the damage detection of a transmission tower-line system subjected to seismic excitation by using semiactive friction dampers as shown in Figure 17. The semiactive control force *u*(*t*) depends on either the sticking or the slipping state of the damper and it can be written as [94, 95]

(20) $$\begin{array}{c} u\left(t\right)=\{\begin{array}{cc} f_{}^{k}\left(t\right), & \text{if}\;\;\left|f_{}^{k}\left(t\right)\right|<\left|f_{}^{d}\left(t\right)\right|\;\;\left(\text{sticking}\right),\\ f_{}^{d}\left(t\right), & \text{if}\;\;\left|f_{}^{k}\left(t\right)\right|\geq \left|f_{}^{d}\left(t\right)\right|\;\;\left(\text{slipping}\right), \end{array}\\ f_{}^{k}\left(t\right)=k^{d}\left[d\left(t\right)-e\left(t\right)\right], \end{array}$$

in which *k* ^*d*^ is the spring stiffness (brace stiffness) of the semiactive friction damper, *f* ^*d*^(*t*) and *f* ^*k*^(*t*) are the friction force and axial force of a semiactive friction damper, respectively, *d*(*t*) denotes the axial displacement between the two ends of the friction damper, and *e*(*t*) is the slip deformation of the friction damper.

**Figure 18:**
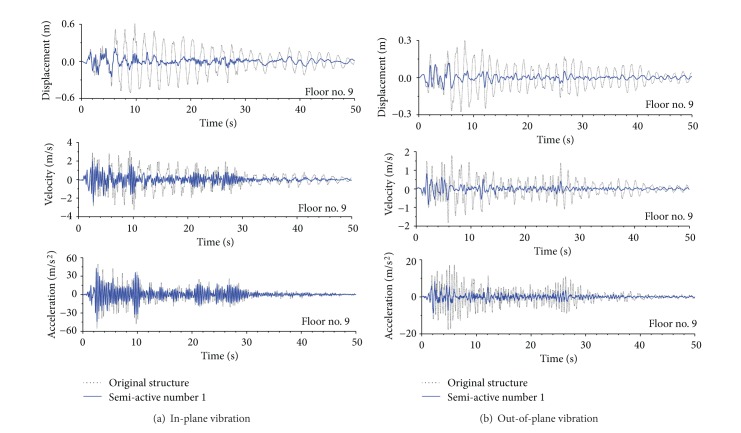
Control performance on top of the transmission tower.

Two semiactive control strategies were proposed for the seismic vibration mitigation. The first one was a clipped-optimal strategy based on fuzzy control principle and the other one was a strategy based on the fixed increment of controllable damper forces. A damage detection scheme was developed in the time domain to identify stiffness damage of the transmission tower. A real transmission tower-line system constructed in China was taken as an example to examine the feasibility and reliability of the proposed vibration control approach and damage detection approach.  Figure 18 indicated the control performance on top of the transmission tower. The results demonstrated that the incorporation of friction dampers into the transmission tower-line system can substantially suppress the earthquake-induced responses of the transmission tower. The damage size and location of the transmission tower can be accurately identified even with noise contamination.

In reality, conventional dynamic design of the transmission-tower line system by using control devices is quite complicated to be carried out by the common structural engineers. To this end, Chen et al. [96] proposed a method for the wind-resistant design of the transmission tower-line system by using viscoelastic dampers. The equivalent damping ratio of the wind-excited transmission tower incorporated with viscoelastic dampers *ζ*_j_* can be determined by

(21) $$\begin{array}{c} \zeta_{j}^{*}=\frac{2\zeta_{sj}\varphi_{j}^{T}K_{S}\varphi_{j}+\eta_{Dj}\varphi_{j}^{T}K_{D}\varphi_{j}}{2\varphi_{j}^{T}\left(K_{S}+K_{D}\right)\varphi_{j}}, \end{array}$$

where *ξ*_*sj*_ is the critical damping ratio of the *j*th mode shape, **φ**_*j*_ is the *j*th mode shape of the controlled tower, and **K**_*S*_ and **K**_*D*_ are the stiffness matrices of the tower and the contribution matrix of viscoelastic dampers to the structural stiffness matrix.

**Figure 19:**
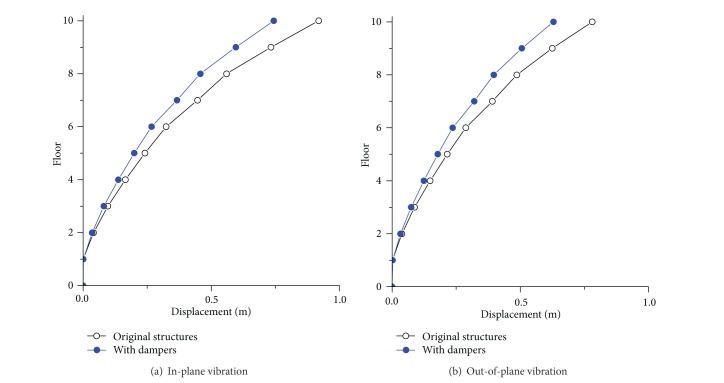
Displacement responses of the transmission tower with/without viscoelastic dampers.

The practical method of the wind-resistant design was developed based on the Chinese design code. A real transmission tower-line system constructed in China was taken as the example to examine the feasibility and reliability of the proposed approach.  Figure 19 displays the displacement responses of the transmission tower with/without viscoelastic dampers. The observations demonstrated that the viscoelastic dampers can be utilized in the wind-resistant design of transmission tower-line system because of its simple configuration as well as satisfactory control performance. The design method proposed can also be applied to wind-resistant design of civil engineering structures installed with other energy-dissipating devices.

**Figure 20:**
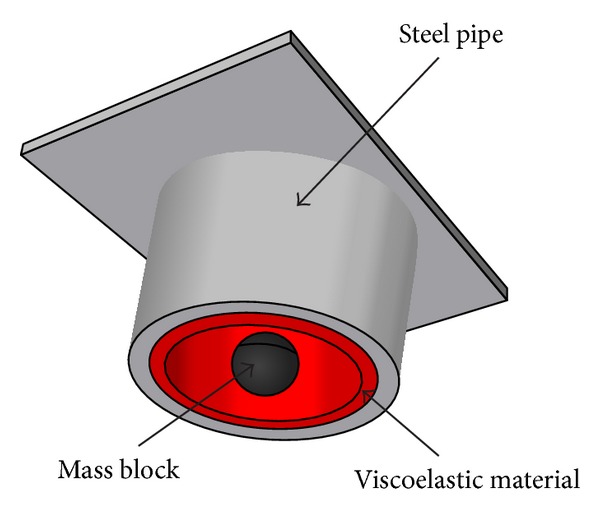
Three-dimensional diagram of a pounding TMD.

Another typical control device commonly utilized in civil engineering structures is the tuned mass damper (TMD). TMD can reduce the structural dynamic responses to some extent, while it requires one or more large additional masses. Owing to the inherent nature of TMD, it can only abate the vibration of tuned mode shapes instead of the global dynamic responses. Tian et al. [97] investigated the seismic control of power transmission tower-line coupled system subjected to multicomponent excitations. The equation of motion of a transmission tower with TMD under multicomponent excitations was established. The structural seismic responses with geometric nonlinearity were computed in the time domain. The optimal design of the transmission tower-line system with TMD was determined based on different mass ratio. The effects of wave travel, coherency loss, and different site conditions on the system without and with control were examined, respectively. More recently, a new type of TMD, the pounding tuned mass damper (PTMD) as shown in Figure 20, was proposed by Zhang et al. [98] to examine the seismic resistant performance of a transmission tower. In the PTMD, a limiting collar with viscoelastic material laced on the inner rim is installed to restrict the stroke of the TMD and to dissipate energy through collision. The pounding force is modeled based on the Hertz contact law, whereas the pounding stiffness is estimated in a small-scale test. A 55 m transmission tower was taken as the example to verify the validity of the PTMD through numerical simulation. Harmonic excitation and time-history analysis demonstrated the PTMD superiority over the traditional TMD.

## 7. Concluding Remarks

An overview is presented in this study on research advances in the analysis of transmission tower-line systems with special emphasis laid upon the response assessment and vibration control. The research activity going on around the world in terms of wind-induced responses, seismic responses, ice effects, and vibration control is reviewed, respectively. It is addressed in this review that analytical approaches based on the transmission tower-line system are promising in comparison with traditional techniques. The approaches based on the tower-line system not only provide reasonable observations, but also have the distinguished superiority in exploring the dynamic interaction between the tower and lines when subjected to dynamic excitations. The investigation of the dynamic performance and control approaches of the transmission tower-line systems is not over yet. There are still difficulties in the researches and the main challenges and future development trends are as follows.

1. Development and improvement of analytical models of tower-line systems are still expected. From the view, it can be seen that recently there have been innovative applications and improvement of the analytical models. Many models for transmission lines have been proposed to simulate the dynamic responses of the line in a more accurate and quick manner with the nonlinearity. Therefore, the analytical models of the tower-line system could be improved accordingly by combining the newly developed cable models with the conventional tower model, which is commonly constructed by using the FE method, to form more powerful models for analyzing structural dynamic responses. Thus, further studies on analytical models are necessary and imperative for the assessment and control of the linear and nonlinear dynamic responses of tower-line systems.
2. Tremendous field measurement demonstrates that the wind loads acting on towers and lines are quite complicated, in particular in the regions close to coastal areas. The loading models and patterns for the extreme wind events, such as typhoon, downburst, and tornado, are quite different to that of common monsoon winds. Up to now, the studies on the loading models of transmission tower-line system subjected to extreme winds are still very limited. The damage, failure, and collapse of transmission towers and lines have been frequently reported. Therefore, wind loading on transmission tower-line system is a practical yet challenging issue that should be investigated in detail in the future.
3. Similar to that of the winds, the loading models and effects of other dynamic excitations such as earthquake and ice shedding still deserve further investigation. The investigation of seismic damages indicates that the dynamic interaction between the truss tower and the soil may be intensive under strong earthquakes. Furthermore, the span of the transmission line is quite large in comparison with common civil engineering structures. Thus, the multiexcitation effects of the transmission tower-line system should be taken into consideration in detail.
4. Transmission lines with long span are prone to the galloping under accumulated snow and ice, which is an important factor to induce the cable rupture and tower failure. The mechanism of galloping and induced instability of the tower-line system is still not clear and the analytical models and approaches for the evaluation on the dynamic stability of tower-line system should be further examined.
5. The widely reported disasters of transmission tower-line systems around the world make it clear that the structures cannot avoid damage and failure under extreme loadings, such as typhoon, downburst, and strong earthquake, even though the system is designed based on the current specifications and codes. The major reason is that the loading patterns specified in the codes cannot depict the extreme loadings and the design method is performed based on static analysis instead of nonlinear dynamic analysis on the interaction of tower-line systems. Accordingly, reasonable methods for the performance assessment of the transmission tower-line system deserve further investigation.
6. The experiment and field measurement are considered as a promising and powerful approach in the performance assessment of transmission tower-line systems. Comparative studies of testing observations with those from the theoretical computation and numerical simulation are limited and needed to be more conducted and addressed. It is found that the tested dynamic properties of the transmission tower are commonly different to those based on the finite element model. This is a practical yet difficult issue, while the model updating methods of transmission tower-line systems have not been reported. Therefore, effective model updating approaches are necessary to accurately predict the structural responses.

It is clear that there still exist some shortcomings in the performance assessment and vibration control techniques of the transmission tower-line system. The benefits of the current technology far outweigh the problems of not using them. This is evident by the tremendous amount of contributions from the scientific community for further developing corresponding novel technology in the real application of transmission tower-line systems. To this end, great efforts should be taken to improve the analytical models and approaches in the near further. The manifestation of the performance assessment and vibration control technology of transmission tower-line systems is warmly expected.

## Figures and Tables

**Table 1:** Types of thunderstorm winds in Australia.

Type	Horizontal scale	Duration
Microburst	1–4 kilometers	2–4 minutes
Macroburst	4–10 kilometers	4–30 minutes
Outflows (gust fronts, squall lines)	10–100 kilometers	1–10 hours
